# Abnormal expression and prognostic significance of EPB41L1 in kidney renal clear cell carcinoma based on data mining

**DOI:** 10.1186/s12935-020-01449-8

**Published:** 2020-07-30

**Authors:** Taotao Liang, Siyao Sang, Qi Shao, Chen Chen, Zhichao Deng, Ting Wang, Qiaozhen Kang

**Affiliations:** School of Life Sciences, Zhengzhou University, Zhengzhou, China

**Keywords:** EPB41L1, Kidney renal clear cell carcinoma, Cell adhesion, Prognostic, APP

## Abstract

**Background:**

EPB41L1 gene (erythrocyte membrane protein band 4.1 like 1) encodes the protein 4.1N, a member of 4.1 family, playing a vital role in cell adhesion and migration, which is associated with the malignant progression of various human cancers. However, the expression and prognostic significance of EPB41L1 in kidney renal clear cell carcinoma (KIRC) remain to be investigated.

**Methods:**

In this study, we collected the mRNA expression of EPB41L1 in KIRC through the Oncomine platform, and used the HPA database to perform the pathological tissue immunohistochemistry in patients. Then, the sub-groups and prognosis of KIRC were performed by UALCAN and GEPIA web-tool, respectively. Further, the mutation of EPB41L1 in KIRC was analyzed by c-Bioportal. The co-expression genes of EPB41L1 in KIRC were displayed from the LinkedOmics database, and function enrichment analysis was used by LinkFinder module in LinkedOmics. The function of EPB41L1 in cell adhesion and migration was confirmed by wound healing assay using 786-O cells in vitro. Co-expression gene network was constructed through the STRING database, and the MCODE plug-in of which was used to build the gene modules, both of them was visualized by Cytoscape software. Finally, the top modular genes in the same patient cohort were constructed through data mining in TCGA by using the UCSC Xena browser.

**Results:**

The results indicated that EPB41L1 was down-expressed in KIRC, leading to a poor prognosis. Moreover, there is a mutation in the FERM domain of EPB41L1, but it has no significant effect on the prognosis of KIRC. The co-expressed genes of EPB41L1 were associated with cell adhesion and confirmed in vitro. Further analysis suggested that EPB41L1 and amyloid beta precursor protein (APP) were coordinated to regulated cancer cell adhesion, thereby increasing the incidence of cancer cell metastasis and tumor invasion.

**Conclusions:**

In summary, EPB41L1 is constantly down-expressed in KIRC tissues, resulting a poor prognosis. Therefore, we suggest that it can be an effective biomarker for the diagnosis of KIRC.

## Background

Kidney Renal Clear Cell Carcinoma (KIRC) is the most common subpopulation of kidney cancer [1] and is derived from the proximal uriniferous tubules, often displays an aggressive phenotype, including metastases to distant organs [2]. KIRC has high morbidity and mortality worldwide and is resistant to radiotherapy and chemotherapy [3]. As we all known destruction of cell adhesion is the first step in cancer metastasis and invasion. At present, surgery is the main method for the treatment of KIRC, and the existing targeted drugs show unsatisfactory efficacy and have great side effects. Therefore, there is an urgent need to determine a reliable prognostic biomarker to predict clinical outcomes and help to make decisions about observation, surgery, medication, and conservative treatment for current.

The protein 4.1 family is a cytoskeletal protein characterized by the presence of the FERM domain (ezrin, radixin, and moesin). Cytoskeleton protein 4.1N is a member of the protein 4.1 family (include 4.1R [4], 4.1B [5], 4.1G [6], and 4.1N [7]), and is produced by the EPB41L1 gene. Just like other protein 4.1 family members, 4.1N as a bridge connecting actin cytoskeleton and various transmembrane proteins plays a key role in cell invasion, migration, and adhesion [8]. It has been reported that protein 4.1N plays an important role in the development of tumors such as breast [8] and ovary cancer [9, 10]. For human kidneys, the previous study has shown that 4.1N associated with the cell adhesion molecule CADM1 expresses in distal tubules. CADM1 is a tumor suppressor in kidney cancer, which suggests that 4.1N may be involved in renal tumorigenesis [11]. However, the role of 4.1N in KIRC remains to be fully elucidated.

In this study, we integrated data of cancer gene expression from public database to perform a variety of bioinformatics analyses, involving the comparison of EPB41L1 expression between KIRC and normal kidney tissues, detecting the influences of EPB41L1 expression on KIRC prognosis, and predicting the co-expression genes of EPB41L1 in KIRC. Further, we performed functional enrichment of EPB41L1-related genes, revealing a new target for diagnosis and treatment of KIRC.

## Materials and methods

### mRNA expression analysis

We collected the mRNA expression of EPB41L1 and APP in KIRC through Oncomine platform (http://www.oncomine.org), which is the largest oncogene chip database currently in the world, containing 715 datasets and data from 86,733 samples, constituting a conveniently integrated data mining platform [12]. This analysis based on a series of researches about KIRC, including Higgins Renal, Gumz Renal, Jones Renal, Beroukhim Renal, and Lenburg Renal. The expression of EPB41L1 and APP was compared between KIRC tissues and normal tissues.

### In silico analysis of the 4.1N in normal and KIRC specimens

Protein expression and distribution of the 4.1N in KIRC patience and normal kidney tissues were searched in the HPA database (https://www.proteinatlas.org/) [13].

### KIRC prognosis analysis

The prognosis analysis by Kaplan–Meier (KM) survival curves was shown by the Gene Expression Profiling Interactive Analysis (GEPIA) (http://gepia.cancer-pku.cn/index.html) database [14].

### Expression of EPB41L1 and APP in various KIRC sub-groups

The UALCAN (http://ualcan.path.uab.edu) is a comprehensive web-portal that allows deeply analyses of TCGA gene expression data. It allows researchers to analyze the relative expression of certain genes in tumors and normal samples, and in various tumor sub-groups such as gender, cancer stages, tumor grade, and other clinicopathological features [15].

### EPB41L1 mutation in KIRC

cBio Cancer Genomics Portal (http://cbioportal.org) is an open-access resource that can be used to interactively explore multidimensional cancer genomics data sets [16]. We used c-BioPortal (http://cbioportal.org) to analyze EPB41L1 mutation in the TCGA KIRC sample. And the “OncoPrint” tab displayed a general view of genetic alterations within each sample in EPB41L1.

### Co-expression gene prediction and GSEA analysis

The differentially expressed genes related to EPB41L1 were screened from the TCGA KIRC cohort (n = 533) through the LinkFinder module in the LinkedOmics database (http://www.linkedomics.org/login.php) [17]. And the correlation of results was tested by the Pearson correlation coefficient. The pathway and network analysis of differentially expressed genes were performed by the LinkInterpreter module, the results of which were signed and ranked, and use gene set enrichment analysis (GSEA) tool to perform analyses of KEGG pathways [18] and GO analysis [containing cellular component (CC), biological process (BP), and molecular function (MF)] [19]. After 500 simulations, P < 0.05 was set as the rank standard.

### Cell line and culture conditions

Human KIRC cell line 786-O were conserved in our laboratory. Cells were cultured in PRIM 1640 medium (Gibco, Carlsbad, CA, USA). The cell media contained 10% fetal bovine serum (FBS, HyClone, Invitrogen), 100 U/ml penicillin and 100 mg/ml, cells were maintained in a humidified incubator at 37 °C with 5% CO_2_.

### Plasmid construction

The purified gene fragment of EPB41L1 was ligated into pEGFP-C3 vector. The ligation products were transformed into *E. coli* DH5α strain competent cells and dispersed onto LB agar plates containing 100 µg/ml kanamycin. After overnight incubation at 37 °C, colonies on the agar plate that contained recombinant plasmids were detected. Electrophoretic separation of all PCR products was performed on 1% agarose gels and sequenced by TSINGKE Biological Technology (Beijing, China). The primers used were: 5′-GCC TCG AGA TGA CAA CAG AGA CAG GC-3′ and 5′-GCG GAT CCT CAG GAT TCC TGT GGC TT-3′.

### Cell transfection

786-O cells were transfected with pEGFP-C3-EPB41L1 and pEGFP-C3 using Lipofectamine 2000 reagent (GenePharma Co., Ltd., Shanghai, China). The plasmid was used to construct a recombinant vector (pEGFP-C3-EPB41L1) which was then transfected into 786-O cells. Meanwhile, 786-O cells transfected with pEGFP-C3 were the negative control group.

### Wound healing assay

For the wound healing assay, 786-O cells were seeded at 2 × 10^5^ cells/well into non-coated 6-well plates (Corning), in PRIM 1640 medium. When cells reached 90–100% confluence, the monolayer was scratched in a straight line with a p200 pipette tip. Debris was removed and the edge of the scratch was smoothed by washing two times with PBS (Life Technologies). Then, the culture medium was replaced and cell migration was monitored. Three fields were selected in each well, which was photographed during 24 h, using the Inverted fluorescence microscope (IX73, OLYMPUS). All samples were assessed simultaneously in three independent experiments. The percentage of the wound covered by migrating cells after 24 h was quantified by ImageJ. The statistical analyses were conducted using the Prism7 (GraphPad Software). Differences were calculated using Student’s unpaired *t* test. All bar graphs were presented as SE and p values less than 0.05 were considered significant.

### RNA extraction and qRT-PCR

Cells were collected, total RNA was prepared using Trizol Reagent (Invitrogen) according to the manufacturer’s instructions. First-strand complementary DNA was synthesized from equal amounts of total RNA (4 μg) using Hifair^®^ III 1st Strand cDNA Synthesis SuperMix for qPCR (11141ES10, TAKARA) and analyzed by Lightcycler 480 SYBR green I master supermix (CW0659S, CWBIO) incorporation in PCR reactions involving specific primers (Table 1) and performed in a real-time qPCR system (Rotor-Gene3000). The expression level was also calculated using the 2^–ΔΔCt^ method.

**Table 1:** qRT-PCR primer sequence

Primer name	Sequence 5′ → 3′
EPB41L1	F: AGGAAACCACGCCGAGACACAA
	R: GGTGGATGAGTTTGCTGTTGGG
GAPDH	F: GGAGCGAGATCCCTCCAAAAT
	R: GGCTGTTGTCATACTTCTCATGG

### UCSC Xena analysis

The heat maps of EPB41L1 and hub genes in the same patient cohort were constructed through data mining in TCGA KIRC by using the UCSC Xena browser (http://xena.ucsc.edu/) [20, 21].

### Gene correlation analysis in GEPIA

Based on the specific data group expressed in the TCGA dataset, we analyzed the correlation between the expression of both EPB41L1 and the target genes by Spearman’s rank correlation coefficient in GEPIA. The specific parameters were set as follows, the X-axis represented EPB41L1, the Y-axis represented other target genes, and their expression levels in tumors and normal tissues served as variables.

### Establishment of interactive network and modules

The interaction between proteins could be sought through the STRING database, accessed through http://string-db.org, we screened out co-expressed genes with interaction scores greater than 0.4 to establish a protein–protein interactive (PPI) network [22]. Then the PPI network was visualized by Cytoscape software with version 3.4.0 [23], in which we could find the highly connected protein-interactive regions, and cluster them into modules through MCODE plug-in with version 1.4.2 in Cytoscape to prepare for the next analysis.

### Statistical analysis

T test was used for differential expression analysis, Log-rank test was used to indicate statistical significance of survival correlation between groups, the differences were considered significant when P ˂ 0.05.

## Results

### Expression of EPB41L1 in human KIRC

**Fig. 1:**
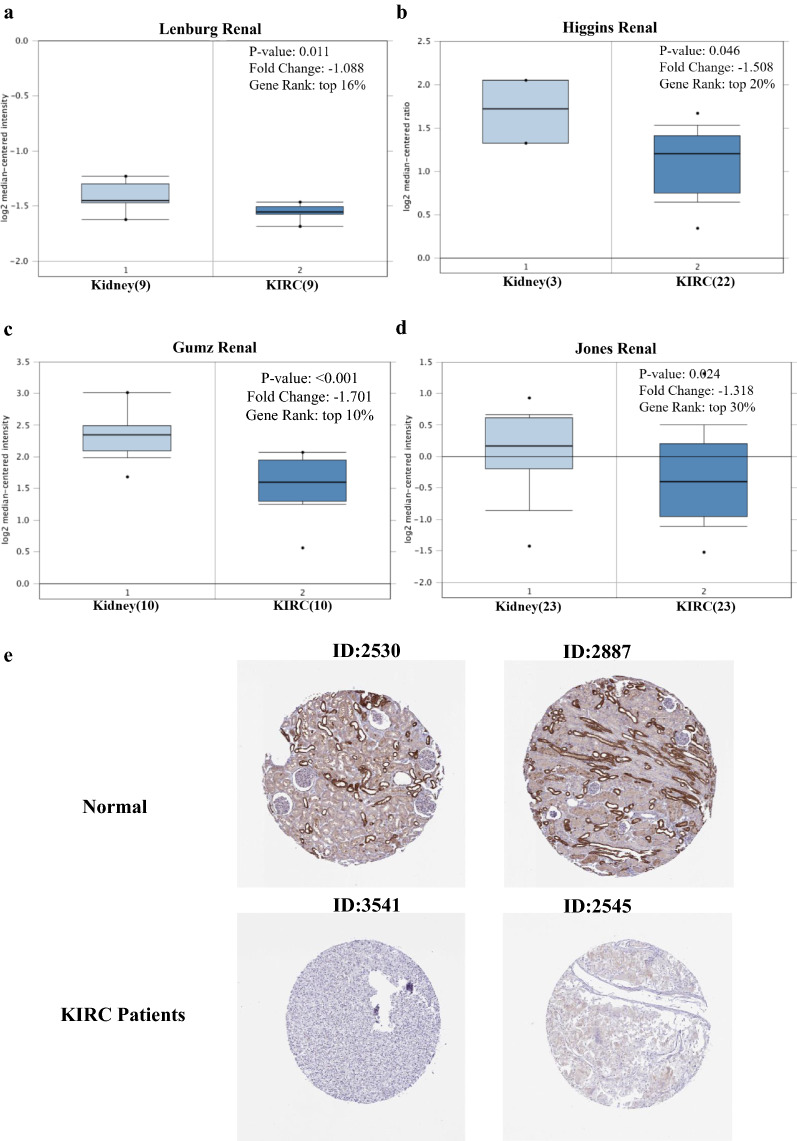
Expression of EPB41L1 in human KIRC. Box plot showing fold change and associated p values based on Oncomine analysis of EPB41L1 levels, respectively in the Lenburg Renal, Higgins Renal, Gumz Renal, and Jones Renal (**a**–**d**). Immunohistochemistry images of proteins 4.1N detected in the HPA database that showed almost negative staining in KIRC tissue but rather a high expression in normal tissue (**e**). Magnification, ×100

Firstly, we analyzed the transcription levels of EPB41L1 in KIRC tumors from a series of studies linked to the Oncomine database and found that the mRNA expression of EPB41L1 in KIRC tissues was obviously lower compared to normal tissues (P ˂ 0.05). As shown in Fig. 1a–d, the mRNA expression of EPB41L1 was among the top 30%, although the differences were not more than twofold between KIRC patients and normal tissues.

We used the HPA database to find normal and KIRC sections from several patients with staining for protein 4.1N. Antibodies used in the HPA database were HPA054104. Immunohistochemistry for the 4.1N in the HPA database showed that protein 4.1N highly expressed in normal cell cytoplasm and plasma membrane but was almost undetectable in KIRC tissue (Fig. 1e).

### EPB41L1 expression in subtype of human KIRC

**Fig. 2:**
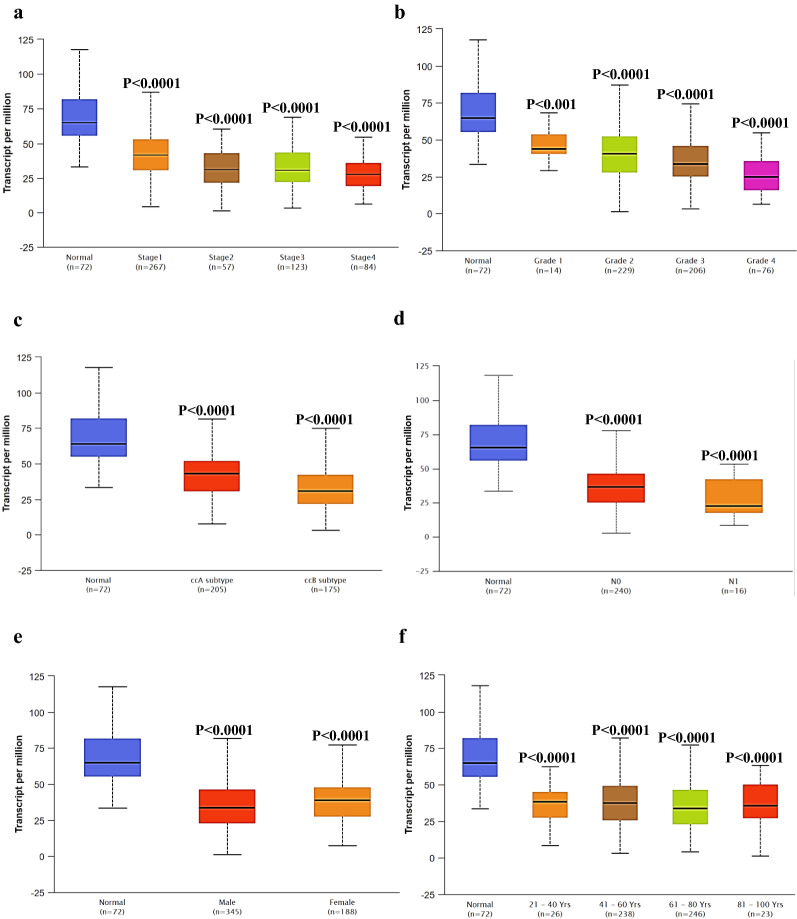
EPB41L1 expression in subtype of human KIRC. EPB41L1 transcription in subgroups of patients with KIRC stratified based on gender, age, and other criteria (UALCAN). Expression of EPB41L1 **a** in normal individuals or KIRC patients in stages 1, 2, 3 or 4; **b** in normal individuals or KIRC patients with grade 1, 2, 3 or 4 tumors; **c** in normal individual of KIRC ccA subtype or ccB subtype in KIRC patients; **d** in normal individuals or KIRC patients with any nodal metastasis status N0 or N1; **e** in normal individuals of either gender or male or female KIRC patients; **f** in normal individuals of any age or KIRC patients aged 21–40, 41–60, 61–80, or 81–100 years

To further prove the specificity of EPB41L1 in KIRC, we integrated various clinic factors of KIRC samples in the TCGA database, for example, cancer stages, tumor grade, KIRC subtype, nodal metastasis status, patients’ gender, and age, and to compare the transcription levels of EPB41L1 in each group. The results showed that KIRC patients, compared with normal subjects, still maintained a low transcription level of EPB41L1 (Fig. 2a–f). Hence, EPB41L1 had the potential to be kidney biopsy-based markers for screening KIRC high-risk patients.

### Down-expression of EPB41L1 predicts poor prognosis of KIRC

**Fig. 3:**
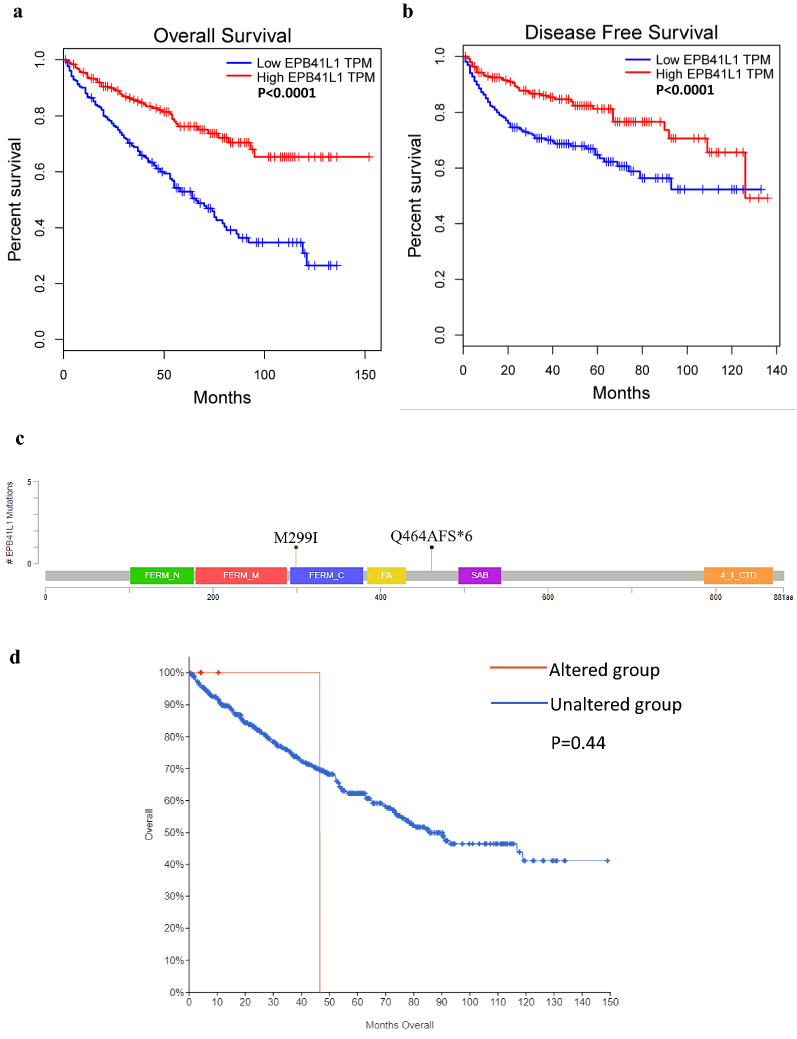
Prognosis and gene alteration of EPB41L1 in KIRC. **a**, **b** KM survival curves for overall survival and disease-free survival in normal and overall KIRC patients by GEPIA. **c** The OncoPrint schematic in c-Bioportal showed that gene alteration of EPB41L1 occurred in 3 (0.6%) of all 537 sequenced cases. **d** KM survival curves between EPB41L1 altered group and unaltered group by c-Bioportal

Then we investigated the prognostic value of EPB41L1 in KIRC. As shown by the KM curve, there was a close relationship between the expression of EPB41L1 and the survival of KIRC patients that the low expression of EPB41L1 caused poor overall survival and disease-free survival (Fig. 3a, b).

### The mutation of EPB41L1 in KIRC and its prognosis

The occurrence of most tumors and the prognosis are related to gene alterations. Here, we evaluated the frequency of EPB41L1 mutations in 537 sequencing data of KIRC patients in the TCGA database through cBioPortal. Result indicated that there were only 3 cases of EPB41L1 mutations (0.6%), one of the mutation sites occurred in FERM_C (M299I), the other one occurred between FA and SAB (Q464AFS*6) (Fig. 3c). But the KM curves analysis of the prognostic value between the EPB41L1 altered group and the unaltered group showed no significant (P = 0.44) (Fig. 3d). This result suggested that the poor prognosis caused by EPB41L1 low expression was not due to its mutation.

### Co-expression genes correlated with EPB41L1 in KIRC

**Fig. 4:**
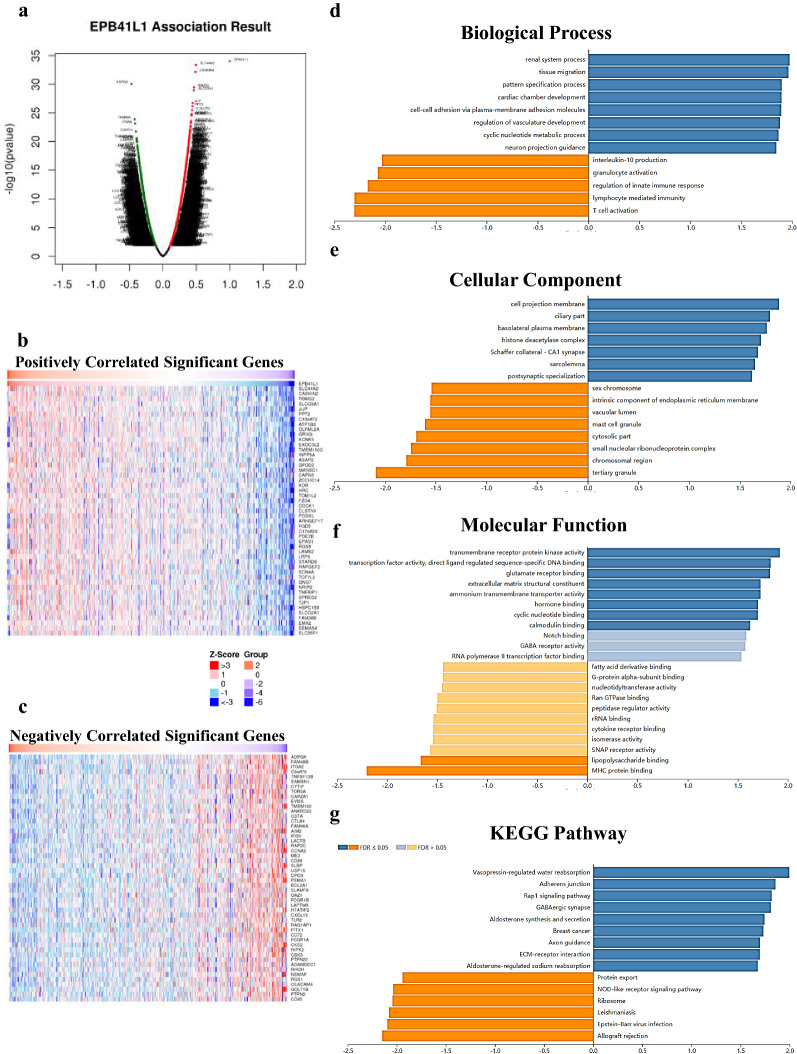
Co-expression genes of EPB41L1 in KIRC (LinkedOmics). **a** A Spearman test was used to analyze correlations between EPB41L1 and genes differentially expressed in KIRC, red indicates positively correlated genes and green indicates negatively correlated genes. **b**, **c** Heat maps show the top 50 significant genes positively and negatively correlated with EPB41L1 in KIRC. The significantly enriched GO annotations and KEGG pathways of EPB41L1 co-expression genes in KIRC were analyzed using GSEA. **d** Biological processes. **e** Cellular components. **f** Molecular functions. **g** KEGG pathway analysis. The x-axis represents the normalized enrichment score, and the y-axis represents the term of GO

We speculated that the role of EPB41L1 in KIRC might be closely related to the function of its neighbor genes in KIRC. We used the LinkedOmics database to analyze the co-expressed genes of EPB41L1 in 533 KIRC cases (Additional file 1). As shown in Fig. 4a, there were 3218 genes represented by dark red dots, having an obviously positive connection with EPB41L1. Conversely, there were 2881 genes, represented by dark green dots, having a notably negative correlation with EPB41L1 (false discovery rate [FDR] < 0.001). 50 significant gene sets were shown by the heat map whether they were positively and negatively correlated with EPB41L1 (Fig. 4b, c).

### GO and KEGG analysis of EPB41L1-related co-expressed genes in KIRC

The outcomes of GO analysis, carried out by GSEA in LinkedOmics, indicated that differentially expressed genes correlated with EPB41L1 were mainly located in cell projection membrane, ciliary part, and basolateral plasma membrane, where they primarily participated in cell tissue migration and cell–cell adhesion. They acted as transmembrane receptor protein kinase activity and extracellular matrix structural constituent (Fig. 4d–f). The functions of these differentially expressed genes were principally enriched in adhesion junction and Rap1 signaling pathway which is a typical cell adhesion-related pathway, through the KEGG pathway analysis (Fig. 4g).

**Fig. 5:**
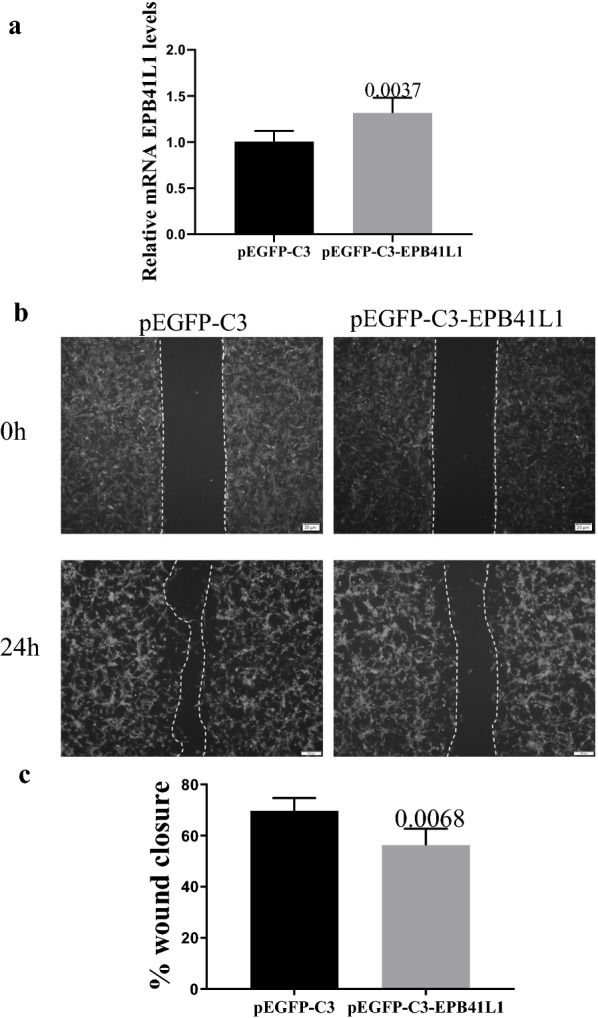
Analysis of 786-O cell migration by in vitro scratch assay. **a** RT-qPCR assessment of EPB41L1 mRNA expression. **b** Micrographs acquired immediately after wounding and at 24 h afterward; **c** Bar graph depicting the rate of cell migration [cell-covered area (%)], at 24 h; data shown are representative of two independent assays and three independent measurements in each. Error bars indicate SE; P values: Student t-test

To confirm the adhesion of EPB41L1 in KIRC, we constructed the EPB41L1 plasmid and transferred it to 786-O cells to overexpress EPB41L1 (Fig. 5a). The results of the wound healing assay showed that in pEGFP-C3-EPB41L1 groups the scratch width was significantly wider than that in the control group (Fig. 5b, c). The results indicated that increasing the expression of EPB41L1 could weaken cell migration.

### Construction of co-expression gene PPI network

**Fig. 6:**
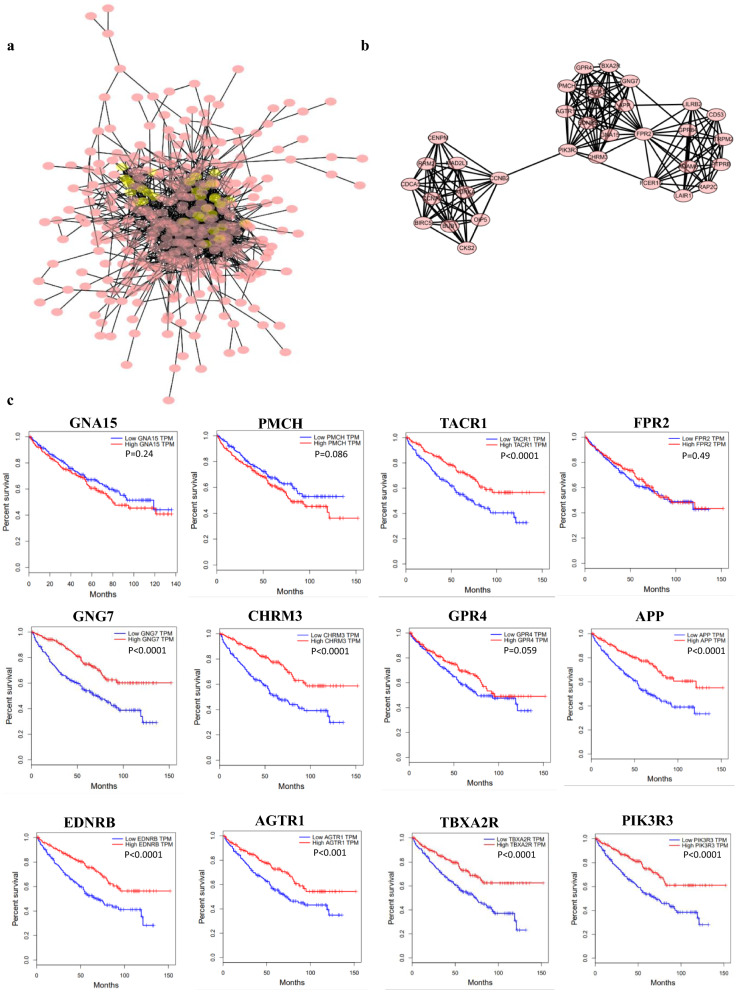
Protein–protein interaction network of co-expression gene (Top500). Protein–protein interaction (PPI) network (**a**) and MCODE analysis (**b**) indicating the hub genes GNG7, PIK3R3, TBXA2R, GPR4, FPR2, TACR1, PMCH, EDNRB, APP, AGTR1, GNA15, and CHRM3 were identified. **c** prognosis analysis of hub genes by GEPIA

The top 500 significantly co-expressed genes were built into a protein–protein network by using the STRING database, and Cytoscape (MCODE plug-in) was used to establish the most important module, highlighted in yellow (Fig. 6a, b). Based on the degree score, the module with the highest score consisting of GNG7, PIK3R3, TBXA2R, GPR4, FPR2, TACR1, PMCH, EDNRB, APP, AGTR1, GNA15, and CHRM3 was identified as potential hub genes (Fig. 6c).

### Prognostic analysis of Hub gene in KIRC

**Fig. 7:**
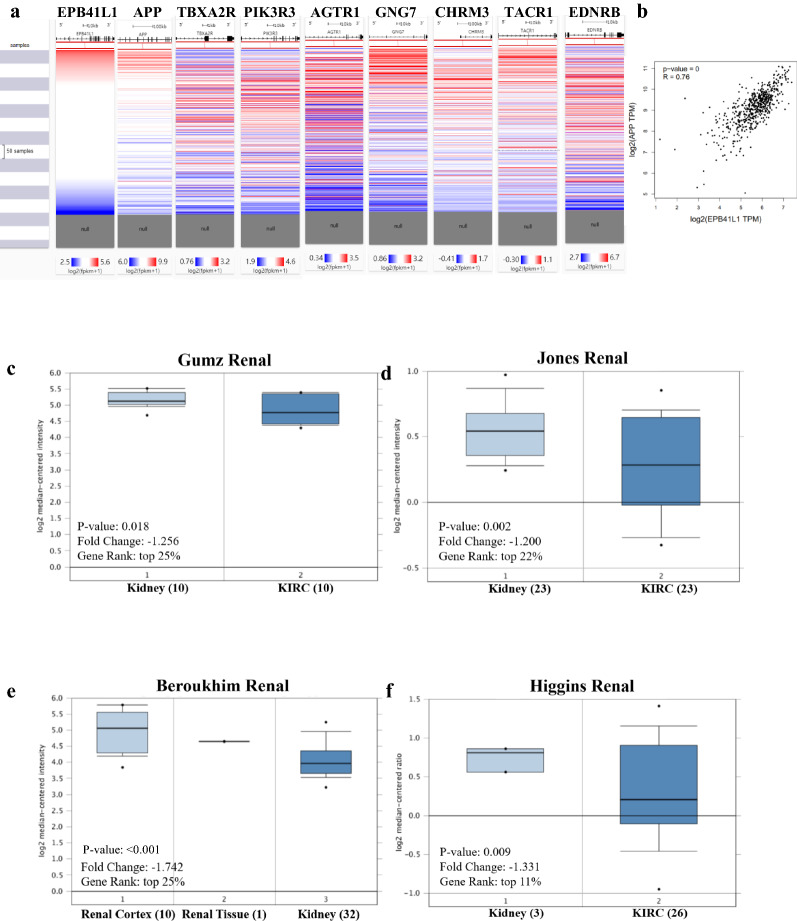
Analysis of EPB41L1 and hub genes in KIRC. **a** The hierarchical clustering of hub genes was constructed using UCSC online database. **b** Correlation between EPB41L1 and APP mRNA expression in KIRC determined using GEPIA. **c** Box plot showing fold change and associated P values based on Oncomine analysis of APP levels, respectively in the Beroukhim Renal, Higgins Renal, Gumz Renal, and Jones Renal

The overall survival of hub genes in KIRC was analyzed by GEPIA database, which indicated that the 8 genes (including APP, TBXA2R, PIK3R3, AGTR1, GNG7, CHRM3, TACR1, and EDNRB) displayed a severe decline in the overall survival rate in low expression groups (Fig. 5c). Next, we used the UCSC Cancer Genomics Browser to hierarchically cluster these 8 hub genes with EPB41L1 and found that the expression pattern between EPB41L1 and APP gene was consistent (Fig. 7a). And there was a high correlation coefficient between EPB41L1 and APP through GEPIA analysis (Spearman’s correlation = 0.76) (Fig. 7b). Therefore, APP might be the most attractive target in cell migration and adhesion among hub genes.

### APP expression in KIRC patients

**Fig. 8:**
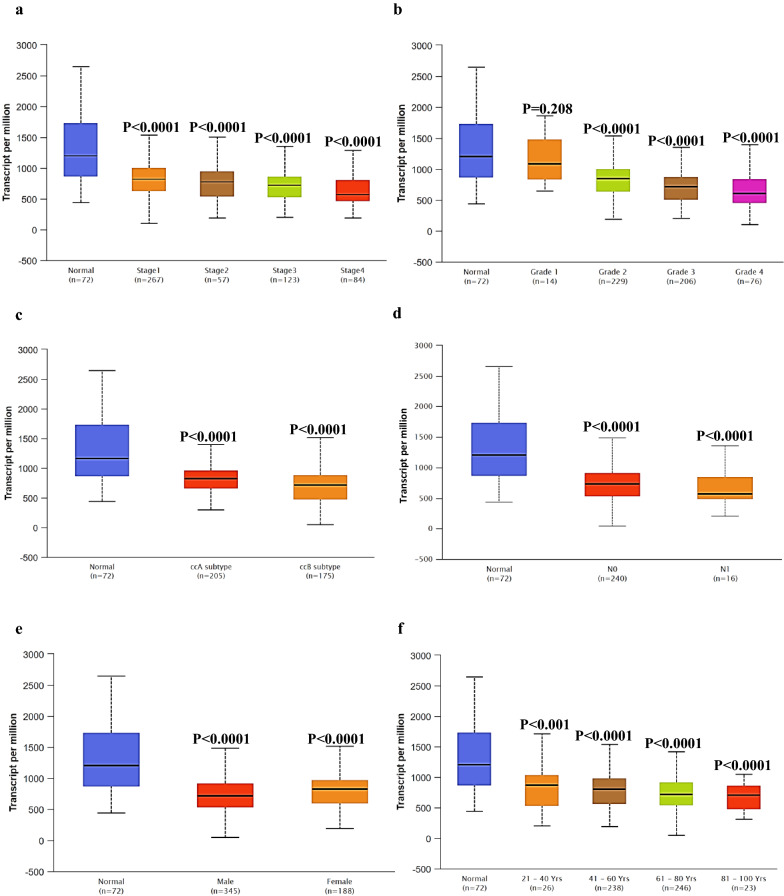
APP transcription in subgroups of patients with KIRC. Expression of APP (**a**) in normal individuals or KIRC patients in stages 1, 2, 3 or 4; **b** in normal individuals or KIRC patients with grade 1, 2, 3 or 4 tumors; **c** in normal individual of KIRC ccA subtype or ccB subtype in KIRC patients; **d** in normal individuals or KIRC patients with any nodal metastasis status N0 or N1; **e** in normal individuals of either gender or male or female KIRC patients; **f** in normal individuals of any age or KIRC patients aged 21–40, 41–60, 61–80, or 81–100 year

Subsequently, we screened a series of datasets from the Oncomine database, such as Gumz Renal, Jones Renal, Beroukhim Renal, and Higgins Renal, to identify the expression of APP in KIRC (Fig. 7c–f). The expression of APP at cancer stages, tumor grades, KIRC subtype, nodal metastasis status, patients’ gender, and age, and in the TCGA database showed that the down-regulation of APP expression nearly existed in all subtypes of KIRC (Fig. 8a–f).

## Discussion

4.1N is a member of the 4.1 protein family and is involved in cell migration and adhesion [7]. Increasing research has shown that 4.1N also plays an important role in several cancers [8–10]. For kidney cancer, in a previous study, Nagata et al. found that CADM4 was connected to 4.1B, and CADM1 was connected to 4.1N in human normal kidney. This study also explored the role of 4.1B-related CADM4 in KIRC [11] and prompted us that 4.1 family could play a key role in KIRC. But this study did not reveal the effect of 4.1N in KIRC. Here, we screened out available datasets associated with KIRC from public databases to confirm the function of 4.1N on the oncoming, progression, and prognosis of KIRC. In this study, the coding gene of 4.1N, EPB41L1 expression had maintained a low level with the emergence and development of KIRC, leading to a poor prognosis. This suggested that EPB41L1 could be identified as a potential biomarker on KIRC diagnosis. We also queried the mutation of EPB41L1 in KIRC through the cBioportal database. Only 3 cases were found in the cBioportal database, indicated that the mutation frequency of EPB41L1 was very low. In addition, we screened EPB41L1 mutations in ICGC and UCSC Xena databases, including only 2 cases (Additional file 2: Figure S1). Comparing these altered groups with the unaltered group, it was found that there was no significant difference in the prognosis between them. We predicted that this result might be due to the small sample size. If more mutation samples are available in future, more interesting results can be obtained. Finally, the co-expression genes related to EPB41L1 in KIRC was analyzed through the Linkedomics database. GSEA function indicated that co-expression genes participated in cell adhesion via GO and KEGG pathway analysis and the wound healing assay results confirmed that EPB41L1 could inhibit cell migration. The APP selected from the co-expression genes could be an EPB41L1 partner in KIRC to regulate the development of KIRC.

KIRC is characterized by a relatively large malignant tumor and easily evolves into distant metastases [2]. The clinical diagnosis of KIRC at early stage is a severe challenge. At present, B-ultrasonography, computed tomography, and nuclear magnetism have been used for clinical diagnosis of KIRC, for patients with localized tumors, tumor recurrence after nephrectomy remains a high risk. The specific molecular target drug TKI is beneficial to metastatic KIRC patients [24]. Unfortunately, due to the inherent or acquired resistance of tumor cells, patients with KIRC will still develop TKI resistance and lead to poor clinical progress [25]. Thus, there is still no effective early-stage diagnosis marker. In addition, considering KIRC is insensitivity to radiation therapy and chemotherapy, surgical resection has become the main treatment method. However, patients with metastatic cancer usually miss the optimal operation time during treatment. Therefore, there is an urgent need to solve the problem of finding potential diagnostic biomarkers in KIRC. Analysis of the expression levels of 4.1N in KIRC clinical cases from TCGA, GEO database, and immunohistochemistry showed that mRNA transcription and protein levels of the target gene in KIRC were significantly lower than normal. The fold change was similar in various KIRC studies. The down-expressed of EPB41L1 occurred in many KIRC cases, which deserved clinical verification as a potential marker in KIRC diagnosis and prognosis.

Cell migration and adhesion are essential for KIRC, and 90% of patient deaths are related to cancer metastasis and invasion [26, 27]. A large number of literature have also associated various cell adhesion and invasion genes with KIRC, such as hypoxia inducible factor 1 (HIF-lα), CD74 [28], CADM4, and 4.1B [11]. In this investigation, EPB41L1 was observed to be differentially expressed in KIRC tissues and adjacent normal tissues, and low expression of EPB41L1 in KIRC predicted poor diagnosis. Therefore, we hypothesized that the poor prognosis caused by the down-expression of EPB41L1 in KIRC might be due to the function of EPB41L1-related genes, which showed that it mainly affected cell adhesion and migration. The function of the neighboring gene network connected to EPB41L1 was closely related to cell adhesion. Therefore, EPB41L1 was the key node of cell adhesion, mainly reflecting on affecting the Rap1 pathway. These results indicated that EPB41L1 was potentially marker on the occurrence and development of KIRC.

The importance of EPB41L1 in the pathogenesis of KIRC was proved from the perspective of prognosis and gene expression. And our study showed that the low expression of EPB41L1 in KIRC had a profound influence on the prognosis and various subtypes of KIRC and cell adhesion. We used a series of bioinformatics tools for neighbor gene analysis of tumor data from public databases. As a result, 4.1N was particularly related to several genes (such as GNG7, PIK3R3, TBXA2R, GPR4, FPR2, TACR1, PMCH, EDNRB, APP, AGTR1, GNA15, and CHRM3).

The human β-amyloid precursor protein (APP), due to its function as a cell surface receptor, participates in numerous biological processes, such as cell growth, adhesion, and axonogenesis [29]. Recently, several pathophysiological functions of APP have been proposed in different human diseases such as neurodevelopmental [30], autism [31], amyotrophic lateral sclerosis [32], multiple sclerosis [33], Alzheimer’s disease [34], and cancer [35–37].

The cooperative regulation of EPB41L1 and APP in cancer cells may indicate a special adhesion mechanism that can synchronize increased cell adhesion based on regulating cell adhesion-related pathway. The down-expression of these two synergistic proteins may be the key mechanism leading to KIRC invasion into other organs. In conclusion, we have confirmed the down-regulation of EPB41L1 and its partner APP in KIRC and verified their importance as prognostic factors. Our research suggests that EPB41L1 may be a promising molecular marker for the diagnosis of KIRC, and provide a new idea for the treatment of KIRC.

## Conclusion

This study systematically integrated public sequencing data to guide the research of EPB41L1 in KIRC. Our work shows that EPB41L1 and its co-expressed gene APP are coordinated to regulated cancer cell adhesion, which can increase the incidence of cancer cell metastasis and tumor invasion and lead to higher mortality in KIRC patients. In general, EPB41L1 may be identified as an important potential biomarker for KIRC diagnosis.

## Supplementary information

**Additional file 1.** The co-expressed genes of EPB41L1 in KIRC.

**Additional file 2: Figure S1.** KM survival curves for overall survival in normal and overall KIRC patients.

## Data Availability

The public datasets used in our work can be found on http://www.oncomine.org, https://www.proteinatlas.org/, and http://www.linkedomics.org/login.php.

## Abbreviations

EPB41L1: Erythrocyte membrane protein band 4.1 like 1
KIRC: Kidney clear cell renal cell carcinoma
TCGA: The Cancer Genome Atlas
GEO: Gene Expression Omnibus
KM: Kaplan–Meier
FDR: False Discovery Rate
GSEA: Gene Set Enrichment Analysis
GO: Gene Ontology
KEGG: Kyoto Encyclopedia of Genes and Genomes
