# Supplementary material for: Abnormal expression and prognostic significance of EPB41L1 in kidney renal clear cell carcinoma based on data mining
Source: Cancer Cell Int. 2020 Jul 30;20:356. doi: 10.1186/s12935-020-01449-8 (PMC7393885; doi:10.1186/s12935-020-01449-8)
Supplement: Supplementary file 2 — Additional file 2: Figure S1. KM survival curves for overall survival in normal and overall KIRC patients. [file 12935_2020_1449_MOESM2_ESM.pptx]

## Slide 1
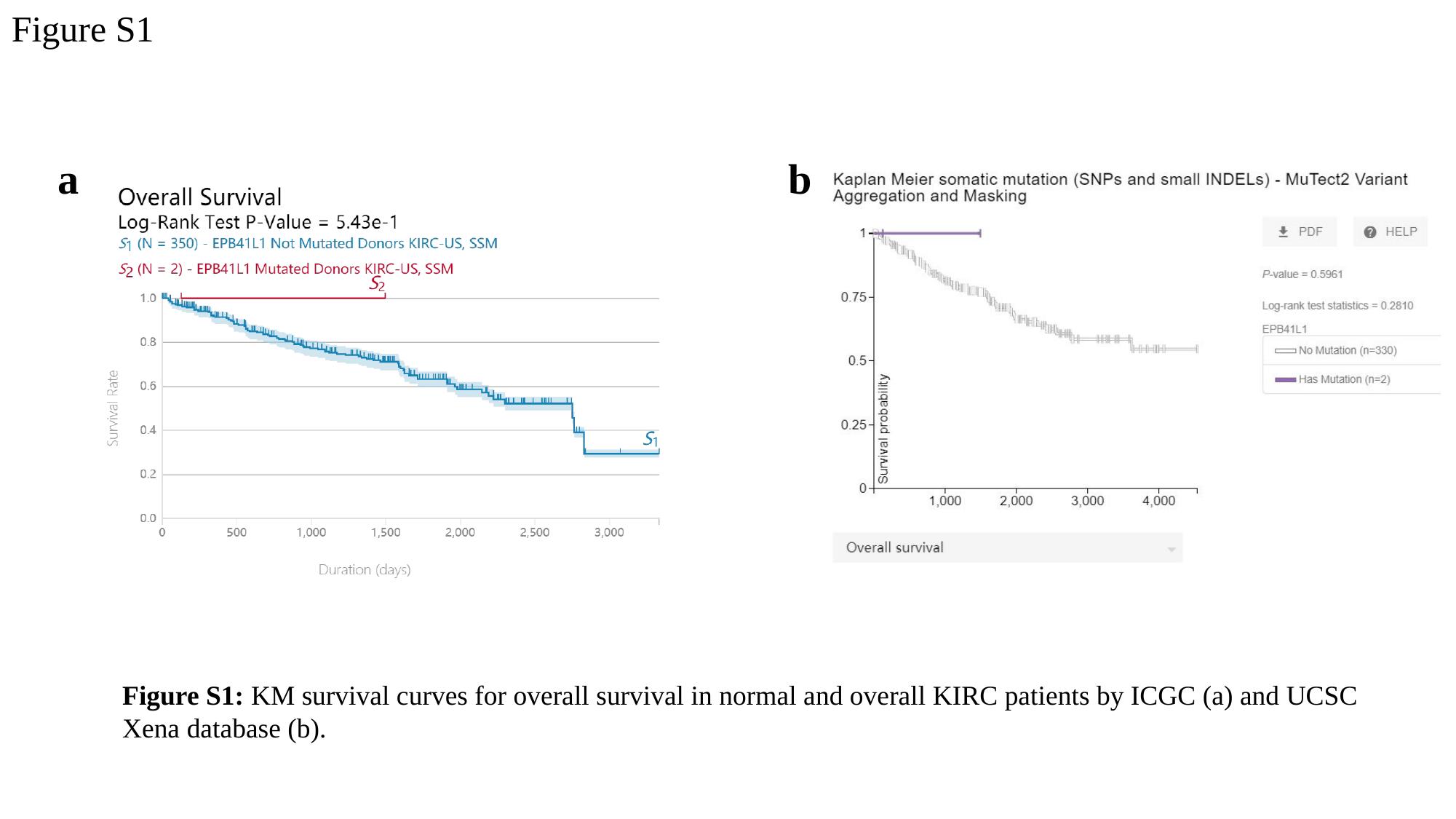

Figure S1
a
b
Figure S1: KM survival curves for overall survival in normal and overall KIRC patients by ICGC (a) and UCSC Xena database (b).
